# NADH Reductive Stress and Its Correlation with Disease Severity in Leigh Syndrome: A Pilot Study Using Patient Fibroblasts and a Mouse Model

**DOI:** 10.3390/biom15010038

**Published:** 2024-12-31

**Authors:** Tamaki Ishima, Natsuka Kimura, Mizuki Kobayashi, Chika Watanabe, Eriko F. Jimbo, Ryosuke Kobayashi, Takuro Horii, Izuho Hatada, Kei Murayama, Akira Ohtake, Ryozo Nagai, Hitoshi Osaka, Kenichi Aizawa

**Affiliations:** 1 Division of Clinical Pharmacology, Department of Pharmacology, Jichi Medical University, Shimotsuke 329-0498, Japan; 2 Department of Pediatrics, Jichi Medical University, Shimotsuke 329-0498, Japan; 3 Biosignal Genome Resource Center, Institute for Molecular and Cellular Regulation, Gunma University, Maebashi 371-8512, Japan; 4 Viral Vector Core, Gunma University Initiative for Advanced Research (GIAR), Maebashi 371-8511, Japan; 5 Center for Medical Genetics, Chiba Children’s Hospital, Chiba 266-0007, Japan; 6 Diagnostics and Therapeutics of Intractable Diseases, Intractable Disease Research Center, Graduate School of Medicine, Juntendo University, Tokyo 113-8431, Japan; 7 Department of Clinical Genomics, Faculty of Medicine, Saitama Medical University, Moroyama 350-0495, Japan; 8 Department of Pediatrics, Faculty of Medicine, Saitama Medical University, Moroyama 350-0495, Japan; 9 Jichi Medical University, Shimotsuke 329-0498, Japan; 10 Clinical Pharmacology Center, Jichi Medical University Hospital, Shimotsuke 329-0498, Japan; 11 Division of Translational Research, Clinical Research Center, Jichi Medical University Hospital, Shimotsuke 329-0498, Japan

**Keywords:** LC-MS/MS, NADH, reductive stress, Leigh syndrome, mitochondrial diseases, *Ndufs4*-KO mice

## Abstract

Nicotinamide adenine dinucleotide (NAD) is a critical cofactor in mitochondrial energy production. The NADH/NAD^+^ ratio, reflecting the balance between NADH (reduced) and NAD^+ (^oxidized^)^, is a key marker for the severity of mitochondrial diseases. We recently developed a streamlined LC-MS/MS method for the precise measurement of NADH and NAD^+^. Utilizing this technique, we quantified NADH and NAD^+^ levels in fibroblasts derived from pediatric patients and in a Leigh syndrome mouse model in which mitochondrial respiratory chain complex I subunit *Ndufs4* is knocked out (KO). In patient-derived fibroblasts, NAD^+^ levels did not differ significantly from those of healthy controls (*p* = 0.79); however, NADH levels were significantly elevated (*p* = 0.04), indicating increased NADH reductive stress. This increase, observed despite comparable total NAD(H) levels between the groups, was attributed to elevated NADH levels. Similarly, in the mouse model, NADH levels were significantly increased in the KO group (*p* = 0.002), further suggesting that NADH elevation drives reductive stress. This precise method for NADH measurement is expected to outperform conventional assays, such as those for lactate, providing a simpler and more reliable means of assessing disease progression.

## 1. Introduction

Next-generation DNA sequencing has revolutionized the molecular genetic diagnosis of mitochondrial diseases [1]. Clinical management remains impeded by an incomplete understanding of the biochemical underpinnings and the absence of quantitative biomarkers for tracking disease progression [2,3]. In patients with mitochondrial encephalomyopathy, lactic acidosis and stroke-like episodes (MELAS), biochemical assays frequently reveal dysfunction of mitochondrial respiratory chain complex I, an NADH:ubiquinone reductase (EC 1.6.5.3), as well as its NADH-reduced equivalents [4,5]. This dysfunction leads to an elevated NADH/NAD^+^ ratio, a phenomenon referred to as NADH reductive stress, which in turn triggers accumulation of conjugated metabolites such as lactate [6,7]. Elevated levels of lactate or lactate/pyruvate have also been observed in Leigh syndrome [8]. Consequently, lactate has historically been employed as a classical, albeit somewhat non-specific, biomarker for mitochondrial disease. However, not all mitochondrial respiratory chain disorders present with hyperlactatemia [9,10], and the degree of lactate elevation does not necessarily correlate with disease severity [11]. Among disorders of the mitochondrial respiratory chain, complex I deficiency (OMIM 252010) is the most prevalent [12,13], with 84% of cases linked to Leigh syndrome (OMIM 256000) [11]. The genetic causes of Leigh syndrome are heterogeneous, but a widely studied mouse model has been developed by knocking out *Ndufs4*, a subunit of complex I [14,15,16]. Sharma et al. suggested that NADH/NAD^+^ reductive stress can serve as a marker of disease severity in mitochondrial disorders [17]. Recently, we introduced a streamlined method for the rapid, precise measurement of NAD(H) (including both NAD^+^ and NADH) [18]. The objective of this study was to employ this method to directly and precisely measure NAD(H) in fibroblasts derived from patients and in a Leigh syndrome mouse model, with the goal of predicting disease severity and enhancing diagnostic accuracy.

## 2. Materials and Methods

### 2.1. Subjects

Fibroblasts were collected from 22 patients diagnosed with mitochondrial disease (Table 1). These samples were obtained with ethical approval from Kanagawa Children’s Medical Center, Chiba Children’s Medical Center, and Jichi Medical University, in accordance with the guidelines of the Ethics Committee of Jichi Medical University. Informed written consent was secured from the parents of all patients.

Ten patients exhibited disorders involving mitochondrial respiratory chain subunits. Of these, seven had mutations related to complex I: one patient harbored a c.[55C>T] mutation in *NDUFA1* (Case 1) [19], another presented with an m.10158T>C mutation in *MT-ND3* (Case 2) [19], whereas three patients carried the m.13513G>A mutation in MT-ND5 (Cases 3) [20], (Case 4) [21], and (Case 5). Additionally, two patients exhibited mutations in *ACAD9*, one with a c.[811T>G];[1766-2A>G] mutation (Case 6) and the other with a c.[1150G>A];[1817T>A] mutation (Case 7) [21]. *NDUFA1*, *MT-ND3*, and *MT-ND5* are subunits of complex I, and *ACAD9* serves as an assembly factor for complex I.

Two patients exhibited mutations associated with complex IV: one with a c.[743C>A] mutation in SURF1 (Case 8) [22] and the other with c.[367_368delAG];[572delC] mutations in *SURF1* (Case 9) [9]. *SURF1* functions as an assembly factor for complex IV.

One patient had an m.8993T>G mutation in *MT-ATP6* (Case 10) [9], a subunit of complex V.

Two patients were diagnosed with MELAS: one carried an m.3243A>G mutation in *tRNA-Leu* (Case 11) [19], whereas the other had an m.5541C>T mutation in *tRNA-Trp* (Case 12) [19].

Two patients were identified with mitochondrial DNA (mtDNA) depletion syndrome: one with c.[451dupC]; [308_310del] mutations (Case 13) [23] and the other with c.[148C>T]; [149G>A] mutations in *MPV17* (Case 14) [23]. *MPV17* is involved in the maintenance of mtDNA integrity.

Two patients were diagnosed with Kearns–Sayre syndrome: one with a single mtDNA deletion (5513 bp deletion; m.8290-13,802) (Case 15) and the other whose genetic testing was inconclusive (Case 16).

One patient had mitochondrial hepatopathy, for which genetic testing was conducted, but the specific mutation remained undetermined (Case 17).

One patient was diagnosed with a short-chain enoyl-CoA hydratase (ECHS1) deficiency, presenting with a heterozygous maternal c.[832G>A] mutation in *ECHS1* (Case 18) [24]. *ECHS1* is involved in valine and fatty acid catabolism in mitochondria.

Two patients exhibited a c.[287A>G]; [287A>G] mutation in *BOLA3* (Case 19) [21], (Case 20). *BOLA3* is involved in the production of iron–sulfur clusters and assembly of the mitochondrial respiratory chain complex.

Additionally, fibroblasts were obtained from two patients with primary CoQ_10_ deficiency. One patient carried biallelic *COQ2* variants (c.[349G>C]; [912+1G>del], Case 21), and the other carried biallelic *COQ4* variants (c.[718C>T]; [421C>T], Case 22) [9].

As controls, fibroblasts from five healthy individuals were purchased from various sources: two from PromoCell (#C-12300, GmbH, Heidelberg, Germany), two from the Japanese Collection of Research Bioresources Cell Bank (#TIG-120, #HT-2020, Osaka, Japan), and one from Lonza Japan (#CC-2509, Tokyo, Japan).

### 2.2. Animals and Sampling

*Ndufs4* knockout mice (B6-*Ndufs4*^tm1^) were generated using CRISPR-Cas9 genome editing at Gunma University, as previously reported [25]. In this experiment, the Cas9 protein and two guide RNAs targeting 5′-ATTCCTAAAGGCTAGCATCA-3′ and 5′-AGTTCAGTACTTGTCATTGG-3′ were used for exon 2 deletion. These components were introduced by electroporation into C57BL/6J (B6)-derived fertilized eggs. The next day, embryos that had developed to the 2-cell stage were transferred into the ampullae of the oviducts of pseudopregnant females. Genotypes of the born mice were confirmed by polymerase chain reaction (PCR) analysis, followed by TA cloning and sequencing analysis. *Ndufs4* homozygotes (*Ndufs4^−/−^*; hereafter, KO) were generated by crossing heterozygotes (*Ndufs4^+/−^*). Five-week-old male whole-body *Ndufs4* KO mice were used in this study, along with age- and sex-matched wild-type controls (*Ndufs4^+/+^*; hereafter, WT). The genotyping of *Ndufs4* alleles was confirmed by PCR analysis of tail biopsy samples.

Brain tissue samples were harvested from the mice, immediately flash-frozen in liquid nitrogen, and stored at −80 °C for subsequent analysis.

### 2.3. Cell Culture and Growth Conditions

Fibroblasts were cultured in low-glucose (1.0 g/L) Dulbecco’s Modified Eagle’s Medium (DMEM) (Thermo Fisher Scientific, Waltham, MA, USA), supplemented with 10% fetal bovine serum (FBS), 100 units/mL penicillin, and 100 μg/mL streptomycin. Cells were incubated at 37 °C in a humidified atmosphere containing 5% CO_2_.

### 2.4. NAD(H) Measurement in Fibroblasts and Mouse Brain

#### 2.4.1. Chemicals and Reagents

β-Nicotinamide adenine dinucleotide (NAD^+^), β-nicotinamide adenine dinucleotide, and reduced disodium salt hydrate (NADH) were purchased from Sigma-Aldrich (St. Louis, MO, USA). Nicotinamide adenine dinucleotide NH_4_ salt [Ribose-^13^C_5_, 98%] (^13^C_5_-NAD^+^) was purchased from Cambridge Isotope Laboratories (Tewksbury, MA, USA). Other solvents were obtained from FUJIFILM Wako Pure Chemical (Osaka, Japan).

#### 2.4.2. LC-MS/MS Conditions

Quantification of NAD(H) in fibroblasts was conducted using a modification of the method described by Ishima and Kimura et al. [18]. Specifically, the following procedures were performed:

NAD(H) concentrations were analyzed using LC-MS/MS (LCMS-8060 NX System, Shimadzu, Kyoto, Japan). For LC analyses, a Waters Atlantis T3 (150 × 2.1 mm, 3 µm) was used. The column oven and autosampler were set to 35 °C and 4 °C, respectively. Mobile phase A consisted of 5 mM ammonium acetate in water, and mobile phase B consisted of 5 mM ammonium acetate in methanol. The flow rate was set to 0.2 mL/min, and the injection volume was 0.2 μL. The gradient program was as follows: 0 to 1 min, 0%B; 1 to 8 min, a linear gradient of 0 to 50%B; 8.1 to 11 min, 90%B; 11.1 to 15 min, 0%B. Probe position was +2.5 mm. NAD^+^ and NADH were detected in ESI-positive mode. MS/MS conditions were as follows: nebulizer gas flow (3 L/min), heating gas flow (10 L/min), interface temperature (300 °C), desolvation temperature (526 °C), heat block temperature (400 °C), and drying gas flow (10 L/min). The collision energies were 46 V for NAD^+^, 19 V for NADH, and 43 V for ^13^C_5_-NAD^+^. NAD^+^, NADH, and ^13^C_5_-NAD^+^ were observed at *m*/*z* 664.0 > 136.1, *m*/*z* 666.0 > 649.2 and 669.0 > 136.2, respectively. NAD(H) concentrations of the samples were quantified by the area ratio with the internal standard reagent (^13^C_5_-NAD^+^) added to the samples. Quality control for NAD(H) measurements in this study is detailed in our previous report [18]. To normalize NAD(H) values, proteins were quantified using an assay kit (#9300A, Takara Bio Inc., Kusatsu, Japan).

### 2.5. Ubiquinone (CoQ) Measurements in Mouse Brain

#### 2.5.1. Chemicals and Reagents

CoQ_10_ was obtained from Tokyo Chemical Industry (Tokyo, Japan), and CoQ_10_-d_9_ was sourced from Iso Sciences (Ambler, PA, USA). CoQ_9_ was acquired from Sigma-Aldrich (St. Louis, MO, USA). Sodium borohydride (NaBH_4_) was purchased from Tokyo Chemical Industry (Tokyo, Japan), and butylated hydroxytoluene (BHT) was supplied by Cayman Chemical (Ann Arbor, MI, USA). Hexane, methanol (LCMS grade), ultrapure water (LCMS grade), and 1-propanol were obtained from FUJIFILM Wako Pure Chemical (Osaka, Japan).

#### 2.5.2. Preparation of CoQ Standard Reagents

Oxidized CoQ_9_ and CoQ_10_ were each dissolved in hexane at a concentration of 1 mM, stored at −80 °C, and subsequently diluted to 100 µM in hexane for each analysis. CoQ_10_-d_9_ (internal standard, IS) was prepared in methanol at a concentration of 100 µg/mL, stored at −80 °C, and diluted to 10 µg/mL in methanol prior to each analysis.

Reduced CoQ_9_ and CoQ_10_ were generated by reducing their oxidized forms using a published method [26], with slight modifications. In brief, 500 µL of 100 µM oxidized CoQ_10_ was added to a hexane-washed glass vial. Subsequently, 5 mg of NaBH_4_, 20 µL of methanol, and 50 µL of water were introduced into the vial. The mixture was vortexed for 3 min and then left in the dark at room temperature for 30 min. Upon completion of the reduction, the reaction was halted by adding 25 µL of 100 µM EDTA, followed by vortexing for 1 min. The sample was then centrifuged at 5000 rpm for 8 min at 4 °C. The upper layer, containing 100 µM reduced CoQ_10_, was transferred to a glass tube. Reduced CoQ_9_ was prepared in the same manner.

Calibration standards containing both oxidized and reduced forms of CoQ_9_ and CoQ_10_ were added to tubes as 100 µL hexane solutions at a concentration of 100 µM each. Following the evaporation of hexane, residues were dissolved in 100 µL of methanol. This mixed standard was then diluted with methanol and used to prepare the calibration standards.

To a 2 mL tube, 10 μL of the mixed standard solution, 25 μL of 5 mg/mL BHT solution, 50 μL of IS solution (10 μg/mL), 500 μL of ice-cold methanol, and ice-cold hexane were added. The mixture was stirred thoroughly for 1 min. Following centrifugation at 5000 rpm for 8 min at 4 °C, 50 μL of the supernatant hexane layer was aliquoted into a separate tube. Hexane was evaporated, and 200 μL of 1-propanol was added, followed by stirring for 1 min. After a second centrifugation (5000 rpm, 8 min, 4 °C), the supernatant was transferred to a vial and used as the analytical sample. All steps were conducted on ice to prevent sample degradation.

#### 2.5.3. Preparation of Mouse Brain Samples

Brain tissue samples were homogenized in a frozen state using a freeze grinder (SK Mill-200, Tokken, Chiba, Japan). Ground tissue (8–20 mg) was weighed in 2 mL hard tubes kept on ice and homogenized at 6.5 m/s for 5 s, repeated 3 times. During homogenization, 25 µL of 5 mg/mL BHT solution, 50 µL of IS solution (10 µg/mL), 500 µL of cold methanol, 500 µL of cold hexane, and 5 mm stainless steel beads were added. Following centrifugation at 5000 rpm for 8 min at 4 °C, 50 µL of the supernatant hexane layer was transferred to a separate tube. After evaporation of hexane, 200 µL of 1-propanol was added and stirred for 1 min. A second centrifugation (5000 rpm, 8 min, 4 °C) was performed, and the resulting supernatant was transferred to a vial for analysis. All steps were conducted on ice to maintain sample integrity.

#### 2.5.4. LC-MS/MS Conditions

Both oxidized and reduced forms of CoQ_9_ and CoQ_10_ were analyzed using an LC-MS/MS system (LCMS-8060 NX System, Shimadzu, Kyoto, Japan). For liquid chromatography (LC) analysis, a Kinetex XB-C18 column (100 mm × 2.1 mm, 2.6 µm, Phenomenex, Torrance, CA, USA) was employed. The column oven was maintained at 40 °C, and the autosampler was set to 4 °C. The mobile phase was 5 mM ammonium formate in methanol. The isocratic flow rate was 0.8 mL/min, with an injection volume of 1 µL, and the total run time was 12 min. Both oxidized and reduced forms of CoQ_9_ and CoQ_10_ were detected in the positive electrospray ionization (ESI) mode. MS/MS conditions were as follows: nebulizer gas flow at 3 L/min, heating gas flow at 10 L/min, interface temperature at 300 °C, desolvation temperature at 526 °C, heat block temperature at 400 °C, and drying gas flow at 10 L/min.

Collision energies were set as follows: 30 V for oxidized CoQ_9_, 31 V for reduced CoQ_9_, 23 V for oxidized CoQ_10_, 37 V for reduced CoQ_10_, and 35 V for CoQ_10_-d_9_.

Transitions used for quantification were as follows: 813 > 197 for oxidized CoQ_9_, 815 > 197 for reduced CoQ_9_, 880 > 197 for oxidized CoQ_10_, 882 > 197 for reduced CoQ_10_, and 890 > 206 for CoQ_10_-d_9_. The concentrations of oxidized and reduced forms of CoQ_9_ and CoQ_10_ in the samples were quantified by calculating the area ratio relative to the IS (CoQ_10_-d_9_) added to the samples.

### 2.6. Statistical Analyses

All statistical analyses were performed using GraphPad Prism (version 7.04 GraphPad Software, Inc., Boston, MA, USA).

## 3. Results

### 3.1. NADH/NAD^+^ Ratio in Fibroblasts from Mitochondrial Disease Patients

**Figure 1:**
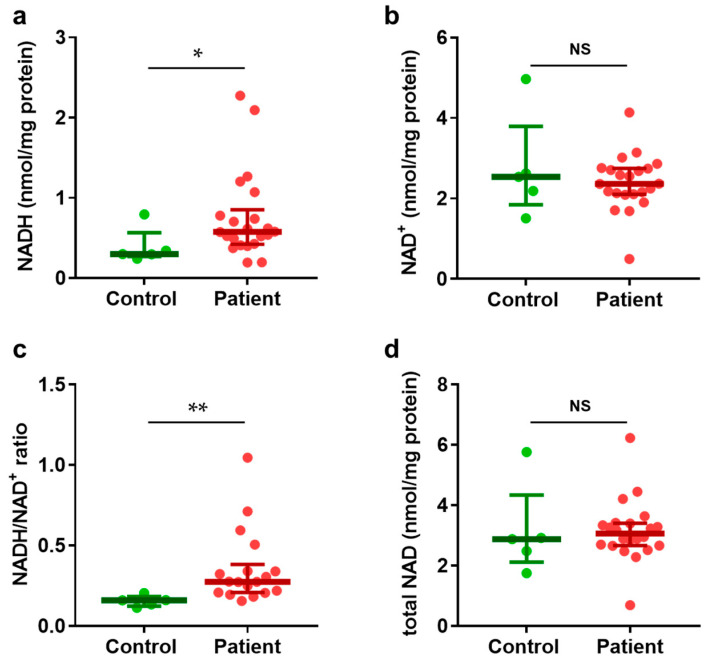
NAD(H) Levels in Mitochondrial Disease Fibroblasts. Comparison of NAD(H) levels in fibroblasts derived from patients with mitochondrial disease (n = 22) and healthy controls (n = 5). (**a**) NADH levels were significantly higher in patients compared to controls. (**b**) NAD^+^ levels remained unchanged in both patients and controls. (**c**) The NADH/NAD^+^ ratio was significantly elevated in patients compared to controls. (**d**) Total NAD(H) levels, representing the sum of NADH and NAD^+^, were comparable between patients and controls. Data are presented as * *p* < 0.05, ** *p* < 0.01; NS = not significant, *p* > 0.05.

We quantified NAD(H) levels in fibroblasts derived from patients with mitochondrial disease. The levels of NADH, NAD^+^, the NADH/NAD^+^ ratio, and total NAD(H) are presented in Figure 1.

NADH levels in the patients were significantly elevated compared to those in the healthy controls (Figure 1a). In contrast, NAD^+^ levels remained unchanged (Figure 1b). Consequently, the NADH/NAD^+^ ratio was significantly increased in the patient group (Figure 1c). Total NAD(H), representing the sum of NADH and NAD^+^, was comparable in the patients and controls (Figure 1d). These results suggest that the elevated NADH/NAD^+^ reductive stress observed in the patient group is attributable to increased levels of NADH, as the total NAD(H) levels were comparable in the two groups. As the NADH/NAD^+^ ratio serves as an indicator of reductive stress [17], these findings are consistent with previous reports [27,28,29].

### 3.2. NAD(H) Levels in Brains of Leigh Syndrome Model Mice

**Figure 2:**
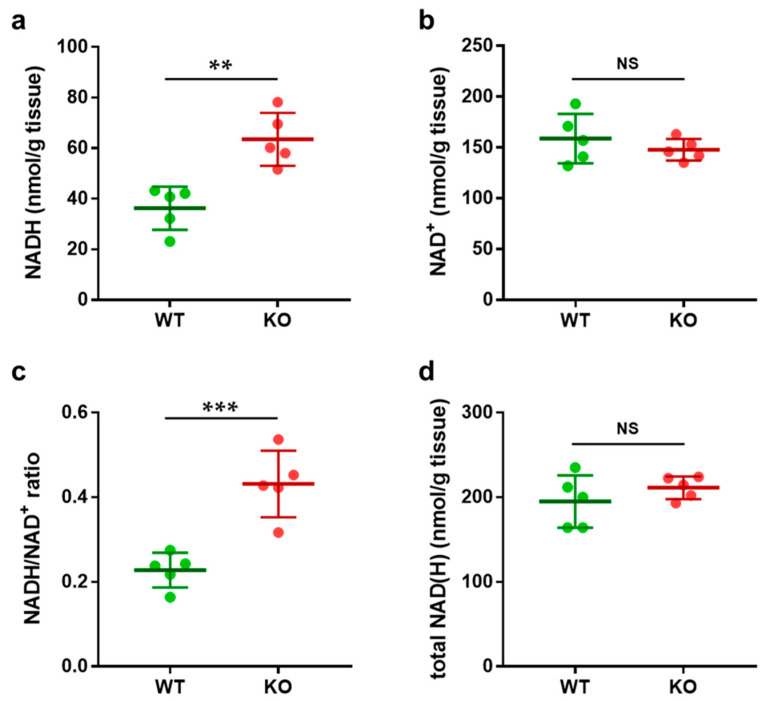
NAD(H) Levels in Mouse Brain Samples. Comparison of NAD(H) levels in brain samples from *Ndufs4* knockout (KO) mice (n = 5) and wild-type (WT) controls (n = 5). (**a**) NADH levels were significantly higher in KO compared to WT mice. (**b**) NAD^+^ levels remained unchanged in both KO and WT mice. (**c**) The NADH/NAD^+^ ratio was significantly elevated in KO compared to WT mice. (**d**) Total NAD(H) levels, representing the sum of NADH and NAD^+^, were comparable between KO and WT mice. Data are presented as ** *p* < 0.01, *** *p* < 0.001, NS = not significant, *p* > 0.05.

Next, we measured NAD(H) levels in brain samples from *Ndufs4* knockout (KO) mice. *Ndufs4* is an 18 kDa protein that forms part of the N-module of complex I, which is essential for ATP production. Deficiency of this protein leads to the clinical phenotype of Leigh syndrome (OMIM 256000) [9,13,30,31,32,33]. The levels of NADH, NAD^+^, the NADH/NAD^+^ ratio, and total NAD(H) are presented in Figure 2.

NADH levels in the KO mice were significantly elevated compared to those in the wild-type (WT) controls (Figure 2a). In contrast, NAD^+^ levels remained unchanged (Figure 2b). Consequently, the NADH/NAD^+^ ratio significantly increased in the KO mice (Figure 2c). Total NAD(H), representing the sum of NADH and NAD^+^, was comparable in the KO and WT groups (Figure 2d). These results suggest that elevated NADH/NAD^+^ reductive stress observed in KO mice is attributable to increased levels of NADH, as total NAD(H) levels were comparable between the two groups.

### 3.3. CoQ Levels in Brains of Leigh Syndrome Model Mice

We also quantified oxidized and reduced forms of CoQ_9_ and CoQ_10_, key components of CoQ in the mitochondrial respiratory chain, in the brains of *Ndufs4* knockout (KO) mice.

**Figure 3:**
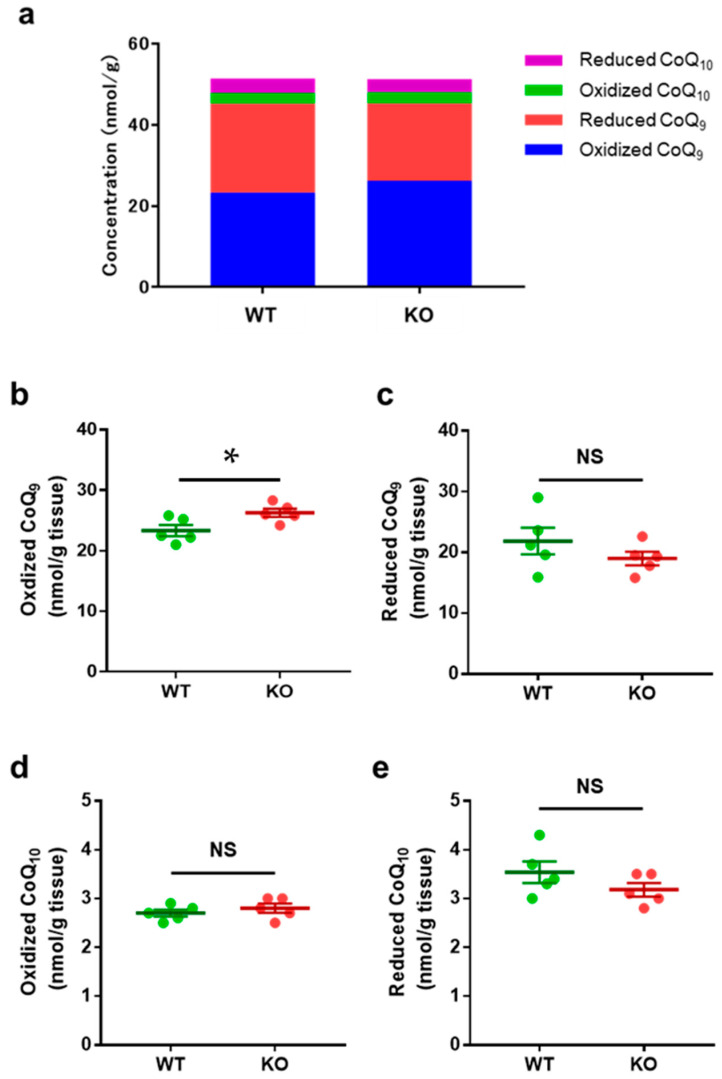
CoQ Levels in Brain Samples from *Ndufs4* Knockout (KO) and Wild-Type (WT) Mice. Comparison of CoQ levels in brain samples from *Ndufs4* knockout (KO) mice (n = 5) and wild-type (WT) controls (n = 5). (**a**) Total CoQ was comparable in WT and KO mice. (**b**) Oxidized CoQ_9_ levels were significantly higher in KO compared to WT mice. (**c**) Reduced CoQ_9_ levels remained unchanged in KO and WT mice. (**d**) Oxidized CoQ_10_ levels showed no difference between KO and WT mice. (**e**) Reduced CoQ_10_ levels were also unchanged in KO and WT mice. Data are expressed as * *p* < 0.05, NS = not significant, *p* > 0.05.

The total CoQ content was similar, with WT at 51.44 nmol/g tissue and KO at 51.26 nmol/g tissue (Figure 3a). When analyzed individually, oxidized CoQ_9_ levels in the KO mice were significantly higher than in the WT mice (Figure 3b) (*p*-value = 0.04). In contrast, reduced CoQ_9_ (Figure 3c), oxidized CoQ_10_ (Figure 3d), and reduced CoQ_10_ (Figure 3e) levels showed no significant differences between the two groups; the *p*-values were 0.22, 0.50, and 0.36, respectively.

## 4. Discussion

### 4.1. NADH/NAD^+^ Ratio in Fibroblasts Derived from Mitochondrial Disease Patients

As noted by Suomalainen et al., there are no non-invasive, specific, and sensitive diagnostic tools for mitochondrial diseases based on serum biomarkers [7]. Consequently, as an indirect measure of NADH accumulation resulting from mitochondrial respiratory chain dysfunctions, the lactate level or the lactate/pyruvate ratio is often used. The first complex in the mitochondrial oxidative phosphorylation (OXPHOS) process, NADH:ubiquinone reductase (EC 1.6.5.3), is critical in electron transport. It is readily understandable that a reduction in the activity of this oxidoreductase, which facilitates the transfer of two electrons from NADH to ubiquinone (CoQ), would lead directly to the accumulation of NADH.

In a study of 15 patients with complex I deficiency, fibroblasts derived from patients exhibited elevated NAD(P)H levels (representing NADH and/or NADPH) compared to those of healthy controls [13]. However, mitochondrial diseases can be caused by a wide range of genetic mutations, and in addition to complex I deficiency, hyperlactatemia and metabolic acidosis can also occur in conditions involving complex IV, complex V (the fifth complex responsible for ATP production), and COQ deficiencies and other mitochondrial gene-related disorders [10,34]. Even so, not all patients with mitochondrial diseases manifest elevated lactate levels. In Japan, 86% of neonatal mitochondrial disease cases exhibit high lactate levels [35]. However, it is estimated that 10–20% of patients with complex I deficiency, which accounts for 80% of mitochondrial diseases, have normal lactate levels [13]. While hyperlactatemia is a key feature of Leigh syndrome, it may not be observed in the early stages of the disease. In some cases, lactate levels are elevated in the brain or cerebrospinal fluid, but not in the blood, meaning that Leigh syndrome cannot be ruled out solely in the absence of hyperlactatemia [34,36].

Mitochondrial dysfunctions are often tissue-specific [37]. Ideally, biospecimens and pathological samples should be obtained from affected organs and tissues; however, in cases such as Leigh syndrome, collecting samples from the central nervous system is challenging. As a result, skeletal muscle and skin-derived fibroblasts have predominantly been used for analysis [33].

In this study, we measured the NADH/NAD^+^ ratio in fibroblasts from 22 pediatric patients with mitochondrial disease using our recently developed method, and the ratio was significantly higher compared to healthy controls (*p* = 0.01). Elevated NADH/NAD^+^ ratios in patient-derived fibroblasts have been previously reported in cases of complex I mutants [27], *MTND1* mutations encoding the complex I subunit [28], and mitochondrial m.3243A>G mutations [29]. However, our findings demonstrate that the NADH/NAD^+^ ratios are also elevated in fibroblast samples, not only with complex I-specific mutations, but also with genetic mutations beyond the commonly studied m.3243A>G mutation. Furthermore, in the present study, NADH levels were significantly elevated in the patient group (*p* = 0.04), whereas NAD^+^ levels and total NAD(H) levels were not different, with *p*-values of 0.79 and 0.31, respectively, suggesting that the high NADH/NAD^+^ ratio resulted from NADH levels.

### 4.2. NAD(H) Levels in Ndufs4-KO Mouse Brain

In the next phase of this study, we measured NAD(H) levels in *Ndufs4*-KO mice, a well-established animal model for studying typical mitochondrial diseases [14], using the same method. Terburgh et al. suggested the possibility of NADH accumulation due to complex I inactivation in skeletal muscle studies with *Ndufs4*-KO mice [38]. However, there are relatively few reports measuring NADH/NAD^+^ ratios in brain samples from *Ndufs4*-KO mice. Previous studies have reported low NAD^+^/NADH ratios [16,39], which were attributed to decreased NAD^+^ levels and increased NADH levels.

The results of this study showed high NADH/NAD^+^ levels, but NAD^+^ was unchanged and only NADH was elevated.

A possible difference from previous reports lies in the measurement methods used. In prior studies, a commercially available assay kit was employed using an amino acid—rather than NAD(H)—as an internal standard. In contrast, our method begins by inactivating proteins with cold methanol, followed by direct measurement of NAD(H) using the stable isotope ^13^C_5_-NAD^+^ as the internal standard. While commercially available kits are relatively inexpensive and easy to use, they do not directly quantify NAD^+^. Instead, these kits rely on a colorimetric determination of total NAD(H), followed by heating or pH adjustment to inactivate NAD^+^, after which the remaining NADH is subtracted from the total NAD(H) to estimate NAD^+^ levels. Additionally, these kits do not initially suppress enzyme activity, and the acidic conditions of the assay can lead to the progressive degradation of NADH, preventing accurate representation of NAD(H) levels [40,41].

Another difference may be the age of the mice used. Yoon’s group and Lee’s group utilized mice 8–10 weeks of age, whereas we employed 5-week-old mice [16,39]. A decrease in NAD^+^ levels with age cannot be ruled out as a contributing factor [42]. Lee et al. reported that brain NAD^+^ levels in *Ndufs4*-KO mice at 9–10 weeks of age were lower than those in wild-type (WT) mice, but at 7 weeks of age, NAD^+^ levels in both groups were comparable [39].

In the present study, we used 5-week-old mice to model mitochondrial diseases in children. Although *Ndufs4*-KO mice are small, they appear healthy until around 5 weeks of age, after which they begin to exhibit symptoms such as ataxia, and they have a median lifespan of 55 days [15,30,32]. This study is the first to report precise measurements of NADH and NAD^+^ levels in the brains of *Ndufs4* KO mice.

### 4.3. CoQ Levels in Ndufs4 KO Mouse Brain

Given that *Ndufs4* is a subunit of NADH reductase and is responsible for transferring electrons from NADH to ubiquinone (CoQ), we measured oxidized and reduced forms of CoQ_9_ and CoQ_10_ in the brains of *Ndufs4* KO mice. Compared to WT mice, there was no significant difference in total CoQ, reduced CoQ_9_, oxidized CoQ_10_, or reduced CoQ_10_ levels. The *p*-values for reduced CoQ_9_, oxidized CoQ_10_, and reduced CoQ_10_ levels were 0.22, 0.50, and 0.36, respectively. However, oxidized CoQ_9_ levels were elevated (*p* = 0.04). In comparisons of ubiquinone (oxidized CoQ), CoQ_10_ is the predominant form in humans, whereas CoQ_9_ is more abundant in mice, as shown in studies of mouse liver samples [43]. In a report on CoQ content in rat brains, a similar rodent model, CoQ_9_ was more abundant than CoQ_10_, with the oxidized form being more prevalent than the reduced form. CoQ_10_ is primarily involved in the Q cycle in complex III, whereas CoQ_9_ is responsible for transferring electrons from NADH and reduced flavin adenine dinucleotide (FADH_2_) to oxidized CoQ_10_ in complexes I and II, respectively [44]. Our study highlights the relationship between complex I and CoQ_9_.

### 4.4. NADH as a Marker of Mitochondrial Disease

Since many patients exhibit elevated lactate levels or lactic acidosis at the onset of mitochondrial disease, lactate and the lactate/pyruvate ratio are currently the primary biomarkers used for diagnosis [45,46]. While these biomarkers have high specificity, they often exhibit low sensitivity [45,47].

There is a correlation between lactate levels measured by cerebral magnetic resonance spectroscopy (MRS) and the occurrence of stroke-like seizures, as well as mortality, in patients with MELAS [48,49]. Similarly, in studies using *Ndufs4* KO mice, brain MRS lactate levels were also associated with mortality [50]. Furthermore, in a study involving 130 patients with Leigh syndrome, elevated cerebrospinal fluid (CSF) lactate levels were correlated with disease severity in patients with an onset age of less than 6 months [36].

In a study utilizing exploratory data analysis techniques on complex I deficiency, fibroblasts from patients in a cluster with earlier disease onset and higher mortality exhibited lower residual complex I activity compared to those from a cluster with later disease onset and lower mortality [51]. Additionally, cells in the early-onset cluster demonstrated more pronounced increases in reactive oxygen species (ROS) and NAD(P)H levels, indicating greater mitochondrial fragmentation [52].

They measured NAD(P)H levels using autofluorescence [51]; however, intracellular NADH is generally more abundant than NADPH [41]. In a study using embryo fibroblasts from *Ndufs4* KO mice, the NAD^+^/NADH ratio was slightly increased compared to wild-type (WT) mice. In contrast, *Ndufs4* gene deletion did not detectably alter the NADP^+^/NADPH ratio [31]. As a result, autofluorescent NAD(P)H is typically assumed to primarily reflect NADH levels. These findings suggest that cellular abnormalities observed in fibroblast models correlate with the clinical phenotypes of mitochondrial disease [53]. Xiao et al. define reductive stress as excessive accumulation of reducing equivalents, such as NADH, beyond what endogenous redox enzymes can manage [6].

Lactate-equivalent metabolites, used as biomarkers, are measured indirectly by assessing lactate accumulation in the cytoplasm. This accumulation occurs after the malate/aspartate shuttle and glycerol-3-phosphate shuttle transfer reducing equivalents, as NADH cannot cross the inner mitochondrial membrane [6,41,54]. NAD(H) is an endogenous molecule, and the redox reaction between NADH and NAD^+^ occurs readily. Moreover, NAD^+^ functions as a cofactor in various cellular processes outside the mitochondria [6,41]. Historically, the inability to accurately measure NAD(H) levels has led to wide variability in reported NAD(H) concentrations [55]. Defining the total amount of NADH and NAD^+^ as the “NAD pool”, studies show that while the NAD pool in the cytoplasm and nucleus is sensitive to oxidative stress, the mitochondrial NAD pool remains stable [6,56]. Thus, changes in NAD(H) levels inside mitochondria do not necessarily reflect those outside. Taken together, the accumulation of NADH itself, rather than the NADH/NAD^+^ ratio, may correlate more closely with disease severity. Therefore, direct measurement of NADH, which is more precise than alternative assessments, such as lactate measurements, would be highly valuable.

Many mitochondrial diseases manifest severe symptoms. An analysis of Leigh syndrome in Sweden revealed that the average time from initial symptom onset to death was approximately 10 months [34]. Another report indicated that disease onset typically occurs between 3 and 12 months of age, with death occurring within 2 years due to disease progression [36]. This underscores the need for prompt diagnosis. We have also demonstrated that NADH can be measured in plasma samples, at least at the animal level, and that there is a correlation between NADH levels in plasma and brain samples from the same individual [18]. If further studies are conducted on clinical samples, NADH could serve as an alternative biomarker to lactate and a potential predictor of disease severity.

### 4.5. Limitations

One limitation of our study is the small sample size, as we only examined a subset of mitochondrial diseases, despite the fact that more than 400 genetic mutations can cause these disorders [1,26]. Additionally, we did not have access to individual blood samples or lactate data for further analysis. While we measured NAD(H) levels in the brains of *Ndufs4* KO mice, this animal model represents only a partial deficiency of complex I; thus, it does not represent the full spectrum of mitochondrial pathologies. Given the variety of NAD-related metabolites, further studies involving both animal models and clinical data from larger cohorts are needed to determine whether NADH levels and NADH/NAD^+^ reductive stress accurately reflect pathogenesis and disease severity.

## 5. Conclusions

This study highlights the potential for precise measurement of the NADH/NAD^+^ ratio using LC-MS/MS as a more accurate biomarker for mitochondrial diseases compared to traditional lactate measurements. Elevated NADH/NAD^+^ ratios were observed in fibroblasts with complex I and other mitochondrial mutations. In the *Ndufs4* KO Leigh syndrome model, high NADH levels in the brain imply a connection between NADH reductive stress and disease severity.

Our findings suggest that precise NADH measurements may not only serve as a clinical biomarker but also assist patient stratification for therapeutic interventions and in monitoring treatment efficacy. Further research is needed to validate NADH as a valuable tool for disease progression assessment and therapy optimization.

## Figures and Tables

**Table 1:** Fibroblast cell lines derived from patients with mitochondrial disease.

Case	Diagnosis	DNA Mutation	Variants, Heteroplasmy Rate ^a^	Function	References
1	Leigh syndrome	*NDUFA1*	c.[55C>T], 100% (X-linked)	Respiratory chain subunits, complex I	[19]
2	Leigh syndrome	*MT-ND3*	m.10158T>C, heteroplasmy (F; 90%)	Respiratory chain subunits, complex I	[19]
3	Neonatal cardiomyopathy	*MT-ND5*	m.13513G>A, heteroplasmy (F; 79%)	Respiratory chain subunits, complex I	[20]
4	Leigh syndrome	*MT-ND* *5*	m.13513G>A, heteroplasmy (F; 26%)	Respiratory chain subunits, complex I	[21]
5	Infantile mitochondrial disease	*MT-ND5*	m.13513G>A, heteroplasmy (B; 77%)	Respiratory chain subunits, complex I	
6	Mitochondrial cardiomyopathy	*ACAD9*	c.[811T>G]; [1766-2A>G]	Respiratory chain assembly factor, complex I	
7	Non-lethal infantile mitochondrial disease	*ACAD9*	c.[1150G>A]; [1817T>A]	Respiratory chain assembly factor, complex I	[21]
8	Leigh syndrome	*SURF1*	c.[743C>A], homoplasmy	Respiratory chain assembly factor, complex IV	[22]
9	Leigh syndrome	*SURF1*	c.[367_368delAG]; [572delC]	Respiratory chain assembly factor, complex IV	[9]
10	Leigh syndrome	*MT-ATP6*	m.8993T>G, homoplasmy	Respiratory chain subunits, complex V	[9]
11	MELAS	*(tRNA-Leu)*	m.3243A>G, heteroplasmy (F; 84%)	Mitochondrial tRNA	[19]
12	MELAS	*(tRNA-* *Trp* *)*	m.5541C>T, heteroplasmy (F; 49%)	Mitochondrial tRNA	[19]
13	mtDNA depletion syndrome	*MPV17*	c. [451dupC]; [308_310del]	Mitochondrial protein synthesis	[23]
14	mtDNA depletion syndrome	*MPV17*	c.[148C>T]; [149G>A]	Mitochondrial protein synthesis	[23]
15	Kearns-Sayre syndrome		Single mtDNA deletion (5513bp del; m.8290-13802)		
16	Kearns-Sayre syndrome		NA ^b^		
17	Mitochondrial hepatopathy		NI ^c^		
18	ECHS-1 deficiency	*ECHS1*	c.[832G>A]	Metabolism of toxic compounds	[24]
19	Mitochondrial cardiomyopathy	*BOLA3*	c.[287A>G]; [287A>G]	Iron–sulfur protein assembly	[21]
20	Mitochondrial cardiomyopathy	*BOLA3*	c.[287A>G]; [287A>G]	Iron–sulfur protein assembly	
21	Primary CoQ_10_ deficiency	*COQ2*	c.[349G>C]; [912+1G>-del]	CoQ_10_ biosynthesis	
22	Primary CoQ_10_ deficiency	*COQ4*	c.[431C>A]; [718C>T]	CoQ_10_ biosynthesis	[9]

^a^: F: fibroblasts; B: blood; ^b^: not administered; ^c^: not identified.

## Data Availability

Datasets generated and/or analyzed during the present study are available from the corresponding author upon request.
